# Extensive SARS-CoV-2 testing reveals BA.1/BA.2 asymptomatic rates and underreporting in school children

**DOI:** 10.14745/ccdr.v49i04a08

**Published:** 2023-04-01

**Authors:** Maria M Martignoni, Zahra Mohammadi, J Concepción Loredo-Osti, Amy Hurford

**Affiliations:** 1 Department of Mathematics and Statistics, Memorial University of Newfoundland, St. John’s, NL; 2 Department of Mathematics and Statistics, University of Guelph, Guelph, ON; 3 Biology Department, Memorial University of Newfoundland, St. John’s, NL

**Keywords:** SARS-CoV-2, COVID-19, testing, asymptomatic, children, underreporting, Newfoundland and Labrador

## Abstract

**Background:**

Case underreporting during the coronavirus disease 2019 (COVID-19) pandemic has been a major challenge to the planning and evaluation of public health responses. School children were often considered a less vulnerable population and underreporting rates may have been particularly high. In January 2022, the Canadian province of Newfoundland and Labrador (NL) was experiencing an Omicron variant outbreak (BA.1/BA.2 subvariants) and public health officials recommended that all returning students complete two rapid antigen tests (RATs) to be performed three days apart.

**Methods:**

To estimate the prevalence of severe acute respiratory syndrome coronavirus 2 (SARS-CoV-2), we asked parents and guardians to report the results of the RATs completed by K–12 students (approximately 59,000 students) using an online survey.

**Results:**

When comparing the survey responses with the number of cases and tests reported by the NL testing system, we found that one out of every 4.3 (95% CI, 3.1–5.3) positive households were captured by provincial case count, with 5.1% positivity estimated from the RAT results and 1.2% positivity reported by the provincial testing system. Of positive test results, 62.9% (95% CI, 44.3–83.0) were reported for elementary school students, and the remaining 37.1% (95% CI, 22.7–52.9) were reported for junior high and high school students. Asymptomatic infections were 59.8% of the positive cases. Given the low survey participation rate (3.5%), our results may suffer from sample selection biases and should be interpreted with caution.

**Conclusion:**

The underreporting ratio is consistent with ratios calculated from serology data and provides insights into infection prevalence and asymptomatic infections in school children; a currently understudied population.

## Introduction

During a pandemic, surveillance is essential for forecasting health care demand and to inform public health decisions. Infection underreporting and inadequate surveillance can lead to unreliable predictions, undermining effective risk assessment ((1)). Underreporting of severe acute respiratory syndrome coronavirus 2 (SARS-CoV-2), which causes coronavirus disease 2019 (COVID-19), has been a major challenge to the analysis of epidemiological data and the implementation of preventive and control measures ((2)). During the pandemic, COVID-19 prevalence has been inconsistently underreported for a variety of reasons, including challenges in maintaining a high-testing capacity ((3)), discouraged testing of non-symptomatic individuals ((4)) and many mild or asymptomatic infections, particularly in children and youth ((5)). Challenges to providing accurate COVID-19 case counts have increased throughout the epidemics. Reasons have been the establishment of more transmissible variants ((6)), the promotion of self-testing alongside no requirement that these results be reported ((7,8)) and increased vaccination coverage, decreasing the likelihood of severe outcomes and the resultant need to seek health care ((9)). All these factors have led to inconsistent variation of case reporting over time, challenging epidemic forecasting.

The Omicron variant of SARS-CoV-2 (formerly BA.1 or B.1.1.529.1, with sister lineage BA.2 or B.1.1.529.2) was first detected in South Africa on November 8, 2021. It was declared a variant of concern by the World Health Organization on November 28, 2021 ((10)). The Omicron variant spread extremely rapidly around the world. In Canada, the first Omicron variant case was reported in Ontario on November 28, 2021, ((11)) and in Newfoundland and Labrador (NL), the first Omicron variant case was reported on December 15, 2022 ((12)). Before the spread of the Omicron variant, there was only limited spread of SARS-CoV-2 in the NL community ((13)). Until that time, NL had implemented a containment strategy, consistent with an elimination (or zero-COVID) strategy ((14,15)). This containment strategy limited SARS-CoV-2 spread through strict border control, contact tracing, self-isolation requirements and non-pharmaceutical interventions aimed to end community transmission whenever outbreaks occurred ((16)).

When Omicron variant infections began spreading in the community, NL reported its highest COVID-19 case counts since the beginning of the pandemic. On January 17,2022, 239 new cases were reported ((17)), which was 0.45% of the provincial population. After January 17, the province no longer publicly reported cases by age group. Until then, 19.7% of the reported cases were for those younger than 20 years of age. A more detailed overview of the epidemiological situation in NL has been published previously ((16)); see also **Supplemental material A**).

With the Omicron variant’s higher transmissibility, its potential to escape the human immune response (meaning that vaccinated individuals and individuals that have already had COVID-19 may be susceptible for reinfection ((18))), and, at the time, unknown health risks, these high-case counts raised concerns of health care capacity overload. The NL elementary (grades K–6), junior high (grades 6–7) and high schools (grades 8–12) closed early for winter break on December 20, 2021 ((19)). To reduce infection spread and protect the health care system, the return to in-person teaching for these students was postponed to January 25, 2022 ((20)).

In addition to the delayed return to school, public health officials strongly recommended that K–12 students (approximately 59,000 individuals) complete rapid antigen tests (RATs; ((4,21))). The Department of Health and Community Services distributed BTNX Rapid Response COVID-19 antigen test kits to schools, and the schools distributed the kits to their students. A first RAT was to be completed on January 22, three days before the return to in-person school. Students testing negative were asked to complete another test the morning of January 25 just before returning to school. Students that recorded positive test results were to self-isolate for 7–10 days, depending on their vaccination status ((22)). At the time, 89.1% of the NL population aged five years and older and 85.7% of the total population were fully vaccinated (defined as two doses) ((23)). Students were to complete these RATs to “reduce the risk of someone attending school while infected” ((16)). There was no requirement to report these RAT results, but positive results could be submitted using the provincial COVID Assessment and Reporting Tool.

The wide distribution of RATs throughout the province, and the recommendation from public health officials for school students to complete these RATs on specific dates, allowed us to study the underreporting of the Omicron variant (BA.1/BA.2 subvariant) and infection prevalence in NL school students. Between February 3 and February 19, 2022, we deployed an internet survey that enabled parents and guardians to voluntarily report the number of positive and negative results for RATs completed by school students (grades K–6 or 7–12) on January 22 and 25. Our survey was unrelated to the provincial COVID Assessment and Reporting tool. Parents were asked to specify whether positive cases were symptomatic or asymptomatic, and to provide the Forward Sortation Area (FSA)—a truncated postal code—and the Regional Health Authority (RHA) where the tests were completed. Results for students in one household were to be reported together (**Supplemental material B**).

The recommendation for school children to complete these RATs first occurred on January 13. However, February 3 was the earliest we could begin the internet survey due to the time it took to obtain the necessary approvals. To ensure informed consent, as many students were younger than 19 years of age (the age of majority in NL ((24))), parents and guardians were asked to report the RAT results, but the reported test results were only for K–12 students. We asked participants to report their FSA (the first three letters/digits of a postal code) so we could determine if spatially adjacent infection spread was occurring, and if there was substantial variation in infection prevalence within and between RHAs. We requested that results be reported together for one household because the Omicron variant is highly transmissible within a household ((25)). Household positivity (rather than individual positivity) is a more reliable measure of prevalence, given that test results from individual students living in the same household are not independent. Furthermore, to estimate underreporting, we compared the results of the RATs with COVID-19 cases reported by the provincial testing system. This comparison was made at the household level because beginning on January 24, 2022, it was stated that household members of COVID-19 cases in NL should not undergo testing at the provincial testing sites ((17)).

Until 2021, COVID-19 testing in Canada occurred mostly for symptomatic individuals, and testing of asymptomatic individuals occurred in vulnerable populations, which included the elderly, residents of long-term care, hospital admissions and, sometimes, contacts of cases. As a less vulnerable population, asymptomatic school children were unlikely to be tested for COVID-19, thus, K–12 students may represent an understudied population. Our analysis aimed to estimate underreporting from the NL provincial testing system, the prevalence and distribution of Omicron variant cases among school students, and the percentage of infections that were asymptomatic for school students that reported positive RAT results.

## Methods

### Survey

Parents and guardians of students in grades K–12 that had completed at least one rapid test on January 22 or January 25 were given the opportunity to answer a web survey to report the test results of their household. Participation was voluntary and consent was required before the survey questions were released. Parents and guardians were told that providing the RAT results would help to understand COVID-19 prevalence and underreporting in NL.

The survey was advertised through broadcast media (two radio morning shows covering eastern NL, two radio morning shows covering central and western NL, and two evening television news shows covering NL) and on social media (Facebook and Twitter). All principals of private and of elementary, junior high and high schools in the NL English School District were emailed requesting that survey participation details be provided to parents and guardians. All Indigenous groups in the province were emailed information describing how to participate in the study. Exceptions were Innu Nation and Sheshatshiu Innu First Nation School, which returned to school later, and requested that their students complete the RATs on different dates.

The survey consisted of four questions, taking approximately five minutes to complete (Supplemental material B). Parents and guardians were asked to provide the following information: 1) the first three letters/digits of their postal code, corresponding to the FSA (e.g. A1A); 2) their RHA (i.e. Eastern Health, Central Health, Western Health or Labrador-Grenfell Health); 3) the number of rapid tests from their household completed on January 22 and January 25, indicating how many rapid tests were negative, positive symptomatic or positive asymptomatic, and 4) whether the students were in grades K–6 or 7–12.

The survey was completed by a total of 1,278 households, where 52% of the households counted more than one student. A total of 2,055 test results were reported (with mostly two-test results per student reported), out of an estimated 59,452 students returning to school, which indicates participation of approximately 3.5%.

Test accuracy: sensitivity, specificity, and confidence intervals

The observed number of positive test results *N*^+^ is the sum of observed positive test results from infected individuals and false positive test results from uninfected individuals, such that:

Equation 1:

*N*^+^ = *p N *σ^+^ + (1 - *p*) *N* (1 - σ^-^) = *N *θ with *p*, σ^+^, σ^- ^∈ [0,1],

where *p* is the true proportion of infected individuals, *N* is the total number of tests, θ is the probability of an individual testing positive, and σ^+^ and σ^−^ are sensitivity (i.e. the probability of testing positive if infected) and specificity (i.e. the probability of testing negative if uninfected), respectively.

By rearranging equation 1, we obtain an estimator p* for the true proportion of K–12 students infected with COVID-19:

Equation 2:

p* = 0 if *N*^+^/*N* < 1 - σ^-^,

p* = (1 - σ^- ^- *N*^+^/*N*)/(1 - σ^-^ - σ^+^) if 1 – σ^-^ < *N*^+^/*N* < σ^+^ ≤ 1,

p* = 1 if *N*^+^/*N* > σ^+^,

and the estimator θ* for the probability of testing positive:

Equation 3:

θ* = p*σ^+^ + (1 – p*)(1 – σ^-^)

Notice that *N*^+^ ∼ Bin(*N*, θ). Therefore, Bin(*N*,θ*) can be resampled to obtain a parametric bootstrap confidence interval estimate.

Sensitivity was estimated as σ^+^=0.9044. This estimate was based on sensitivity values at different viral loads, and on estimates of viral load during infection ((26)). Specificity was assumed to be σ^−^=0.994, based on the study of Parvu *et al.* ((27)). Testing positive if uninfected is very unlikely (with a mean of six out of every 1,000 tests completed), while testing negative if infected can occur with a mean of one out of every 10 cases. A complete derivation of the sensitivity and specificity estimates is provided in **Supplemental material C**.

The observed number of positive asymptomatic cases includes true positive asymptomatic cases and false positive asymptomatic cases, which could be false positive cases (with very low probability, as discussed above ((27))) or positive symptomatic cases falsely reported as asymptomatic. We could not estimate the proportion of symptomatic cases that may be falsely reported as positive asymptomatic, as this is based on participants’ self-assessment; therefore, our analysis of asymptomatic cases is based on the raw reported cases, for which no confidence intervals can be provided.

### Data analysis

Anonymized survey results and the code used for the analysis is publicly available. Each row of the data corresponds to the reporting of a single household, where column entries correspond to the number of positive tests (distinguishing between symptomatic and asymptomatic cases), and negative tests in grades K–6 and grades 7–12.

Our analysis provided insights into the following: 1) the rates of underreporting of COVID-19 cases (Omicron variant, BA.1/BA.2 subvariant) in NL at the population level and at the household level; 2) the proportion of positive tests occurring in elementary (primary) schools (grades K–6) and in junior high and high schools (grades 7–12), and the corresponding proportions of asymptomatic cases; and 3) the spatial distribution of positive households in the province.

Test accuracy was taken into account by considering test sensitivity and specificity. Data were analyzed using the programming language R and the Postal Code Conversion File ((28)).

### Underreporting

To gain insights into COVID-19 underreporting, we compared estimates of the percentage of positive tests among K–12 students (obtained using the survey-reported RAT results) with provincial case counts (Supplemental material A, **Figure S1**). Provincial case counts were based on the Public Service Advisory COVID-19 announcements from the Department of Health and Community Services, which reported the daily number of new cases ((29)).

In NL, publicly available daily age-structured provincial case counts ended on January 17, 2022, after which only the total number of new cases was provided. By considering age-structured active cases reported till January 17 we derive the percentage of active cases among the younger age group, consisting of individuals aged 0–19 years old (Supplemental material A, **Figure S2**). We use this percentage to obtain an estimate for reported COVID-19 prevalence among the age group 0–19 years when the rapid antigen testing occurred on January 22 and January 25 (Supplemental material A). We estimated that 0.49% of the NL population and 0.45% of the age group 0–19 years (averaged across January 20 to January 27, 2022) were reported infected with COVID-19. Finally, we use these estimates to quantify the reported household positivity, estimated to be 1.2%. A discussion of the comparison between reported COVID-19 prevalence and estimated percent positivity in K–12 students and prevalence of COVID-19 in households, derived from the rapid antigen testing results is provided in a later section of this article.

### Analysis of positive cases

The total number of positive tests was calculated from the number of positive tests on January 25 and the number of positive tests on January 22 that were not subsequently reported on January 25. We defined negative tests as the number of negative tests on January 25. This decision was made because parents and guardians were instructed by public health officials not to carry out a second test if the first test was positive, and we decided this after noting that 69 households (out of 1,278) reported different entries on the first and the second testing date (**Supplemental material D**). Positive cases are reported at the provincial level and were divided into symptomatic and asymptomatic cases. The proportion of reported positive cases in elementary (grades K–6) and junior high and high schools (grades 7–12) was also reported.

### Spatial distribution of cases

We defined positive households as households reporting at least one positive result on either January 22 or January 25. The percentage of positive households was computed at the level of the RHA and for each FSAs, as described later in this article. We performed Moran’s I statistics ((30)) to investigate the correlation between spatial proximity and COVID-19 prevalence rates in different FSAs.

## Results

### Underreporting

When considering the survey-reported RAT results for K–12 students, we estimated that 5.0% (95% CI, 3.8–6.5) of households were positive for COVID-19. When considering the provincial COVID-19 data, we estimate that 1.2% of all households were positive for COVID-19, if we assume that only one test per household was reported in a single day. When comparing our estimates with the provincial estimates we determined that the number of underreported positive households was 4.3 (95% CI, 3.1–5.3) times higher than the counts reported by the NL testing system.

The RAT results that we collected at the individual level indicate a percent positivity of 3.7% (95% CI, 2.9–4.7) among children and youth (**Table 1**). Provincial reporting was lower, at 0.45% (Supplemental material A) indicating that on average only one out of every 8.4 (95% CI, 6.4–10.4) infections has been reported, although we note that this calculation overlooks that infections spread more readily to other household members than to members of the wider community.

**Table 1:** Rapid antigen test results at the provincial level and at the level of the four Regional Health Authorities of Newfoundland and Labrador

Region	Total reported positive tests	Total tests	Percent estimated true positives (95% CI)	Total reported positive households	Total reporting households	Percent estimated positive households (95% CI)
Newfoundland and Labrador	82	2,055	3.7% (2.9–4.7)	66	1,278	5.0% (3.8–6.5)
Eastern Health (EH)	61	1,648	3.5% (2.5–4.5)	46	1,019	4.4% (3.0–5.8)
Central Health (CH)	5	105	4.6% (3.9–9.9)	5	63	8.2% (1.1–17.0)
Western Health (WH)	11	221	4.9% (1.9–8.4)	10	143	7.1% (2.4–11.8)
Labrador-Grenfell Health (LG)	5	81	6.2% (0.7–13.1)	5	53	9.8% (1.4–18.2)

Abbreviation: CI, confidence interval

### Analysis of positive cases

A total of 82 out of 2,055 tests were reported positive, giving an estimate of the true prevalence as 3.7% (95% CI, 2.9–4.7) (**Table 2**). A larger proportion of these positive tests, namely 62.9% (95% CI, 44.3–83.0), was reported in elementary school students, while the remaining 37.1% (95% CI, 22.7–52.9) was reported in junior high and high school students (grades 7–12). More than half of the cases were reported as asymptomatic (59.8%), with no significant difference in the proportion of asymptomatic cases in grades K–6 and in grades 7–12 (i.e. 60.8% and 58.1% respectively).

**Table 2:** Rapid antigen test results and estimates of Omicron variant positivity and percent asymptomatic infections^a^, Newfoundland and Labrador

Definition	Results (95% CI)
Total reported positives	82
Total reported tests	2,055
Percent estimated true positives	3.8% (2.9–4.7)
Total reported positives (grades K–6)	51
Total reported positives (grades 7–12)	31
Total reported (grades K–6)	1,192
Total reported (grades 7–12)	863
Positives in grades K–6 (percent of total estimated true positives)	62.9% (44.3–83.0)
Positives in grades 7–12 (percent of total estimated true positives)	37.1% (22.7–52.9)
Total reported asymptomatic	49
Total reported asymptomatic (grades K–6)	31
Total reported asymptomatic (grades 7–12)	18
Asymptomatic (percent of total reported positives)	59.8%
Asymptomatic (percent of reported positives in grades K–6)	60.8%
Asymptomatic (percent of reported positives in grades 7–12	58.1%

Abbreviation: CI, confidence interval

^a^ In elementary schools (grades K–6) and in junior high and high schools (grades 7–12)

### Spatial distribution of cases

**Figure 1:**
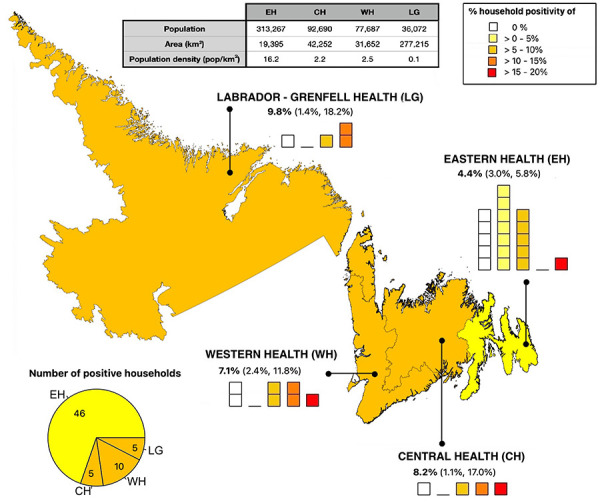
Map of Newfoundland and Labrador, divided by Regional Health Authorities^a^ Abbreviations: CH, Central Health; EH, Eastern Health; LG, Labrador-Grenfell Health; WH, Western Health ^a^ Percent values represent the percentage of positive households in each region, with 95% confidence intervals. The pie chart represents the number of reported positive households for each health region as a fraction of the total number of positive households in the province. The population, area, and population density of each RHA are provided in the table in the top of the figure

A total of 66 out of 1,278 households reported at least one positive test on January 22 or January 25, with corresponding household positivity of 5.0% (95% CI, 3.8–6.5). Reports of positive tests were distributed across all four RHAs. Figure 1 represents a map of NL, divided by RHAs, from left to right, Labrador-Grenfell Health, Western Health, Central Health, and Eastern Health. The household positivity (i.e. the percentage of households which reported positive test results from K–12 students) reported by each of its FSAs is shown for each RHA, where each square corresponds to a single FSA within the RHA and the colour of the square represents the reported household positivity. We include only results of FSAs for which test results of students of six or more households have been reported. All RHAs reported household positivity higher than 10% in one or more FSAs, as well as low or zero positive tests in other FSAs. The FSAs were not identified, because we do not have consent from the participants to release this information. The population size, area and population density of each RHA is provided in Figure 1. The total number of households reporting is provided in Table 1.

Participants from Eastern Health, as the smallest but most populated health region of the province, reported results for 17 out of 18 FSAs. This region reported 46/1,019 positive households (out of 66 total positive households in the whole province), but lower COVID-19 prevalence rates with respect to other health regions, with the percentage of positive households being 4.4% (95% CI, 3.0–5.8). Participants from Central Health reported 5/63 positive households and a household positivity of 8.2% (95% CI, 1.1–17.0), based on the RAT results of four out of seven FSAs. Participants from Western Health reported results from seven out of seven FSAs, with 10/143 positive households and household positivity of 7.1% (95% CI, 2.4–11.8). Participants from Labrador-Grenfell Health reported results from four out of four FSAs, with 5/53 positive households, with a household positivity estimated as 9.8% (95% CI, 1.4–18.2). The number of FSAs reporting low versus high percentage household positivity for each RHA is provided in Figure 1. Because of lower reporting rates and possible sampling biases, there is high uncertainty associated with household positivity in the regions of Labrador-Grenfell Health, Central Health and Western Health, and, more generally, with prevalence at the FSAs level.

We obtained a Moran’s I coefficient of -0.08, and a *p*-value of 0.35, indicating no correlation between spatial proximity and COVID-19 prevalence rates among FSAs.

## Discussion

Underreporting has been a major challenge for COVID-19 pandemic monitoring and response planning. These challenges have increased with the establishment of the highly transmissible Omicron variant ((7,31)) and the expanded use of rapid tests, the results of which are not officially reported in some jurisdictions ((7,8)). Underreporting rates may have been particularly high among children and youth, given their relatively low risk of experiencing severe outcomes ((32)). In NL, public health officials recommended that all K–12 students complete RATs on January 22 and 25, 2022. We conducted an online survey where parents and guardians of K–12 students could report these RAT results. Self-administered rapid tests were not reported in the NL provincial case counts, and characteristics of the NL population eligible for testing under the provincial system ((4)), were very different than the characteristics of the population that completed the RATs on January 22 and 25, 2022. We estimated that only one out of every 8.4 (95% CI, 6.4–10.4) cases occurring in children and youth or one out of every 4.3 (95% CI, 3.1–5.3) positive households, were reported by provincial case counts.

The COVID-19 Immunity Taskforce uses serological analysis of blood donations to estimate the percentage of provincial populations that have been infected with SARS-CoV-2 ((33)). When interpreted relative to the number of cases reported by the NL testing system, these serology data imply that from January to February 2022, one in every 2.3 cases were reported (Supplemental material C, **Table S1**).For comparison, in other Canadian provinces from January to February 2022, the underreporting ratio ranged from one in every 17.2 cases reported (British Columbia) to an equal number of cases reported and detected by serology (Prince Edward Island, **Supplemental material E, Table S2**). Underreporting ratios are generally highest in children ((34)). The underreporting ratio that we estimate from our study of rapid antigen testing in K–12 students is broadly consistent with COVID-19 Immunity Taskforce data. Eligibility for testing, such that the results of the testing could be reported in the provincial case counts, was relatively unrestricted in NL at the time of our study, while in all other provinces except for Prince Edward Island, most individuals were ineligible for testing under the provincial systems due to age restrictions on eligibility (Supplemental material E).

Most of the positive cases occurred in elementary schools (62.9%, 95% CI, 44.3–83.0), while previously published articles found higher COVID-19 prevalence in junior high and high schools (secondary schools) relative to elementary schools ((35–37)), presumably due to student cohorting. Elementary school students tend to remain with the same classmates throughout the day, while older students have different classmates in different classes. However, for the RAT results collected in our study, testing was conducted after schools had been closed for five weeks; therefore, student cohorting or other public health measures aimed to reduce COVID-19 spread in schools would not have impacted our results. Potentially, a major factor influencing our results was the vaccination status of the students. Vaccination rates for 5–11-year-olds in NL were the highest in Canada, with 75% having received one dose of the vaccine on January 19, 2022 ((38)). However, youth aged 12 years or older became eligible for vaccination starting May 23, 2021, while children aged five years and older became eligible for vaccination only on November 23, 2021. At the time of our study (specifically, on January 22, 2022), nearly all junior and high school-aged youths were fully vaccinated (96.7% of NL residents aged 12–17 years), while nearly all elementary school-aged children had not completed a full-vaccination series (only 3.3% of NL residents aged 5–11 years had completed a full-vaccination series ((39))).

Whether children and youth are more susceptible than adults to SARS-CoV-2 infection has been a matter of debate ((40)). Understanding the role that children play in the transmission of the virus is key to inform public health policies for the implementation of non-pharmaceutical interventions, such as school closures. Given the consequences of school closures on mental and social health ((41,42)), it is important to understand the effect that closing schools has on COVID-19 transmission. Understanding the role of school children in SARS-CoV-2 spread may also help inform vaccine prioritization strategies. Possible vaccination strategies include prioritizing essential workers (e.g. teachers or other workers with a large number of social contacts), which would reduce transmission and the total number of infections ((43)).

We estimate that 59.8% of the positive tests were asymptomatic, where asymptomatic rates were similar among elementary school students (60.8%) and students in junior high and high schools (58.1%). Previous studies have reported asymptomatic rates associated with the Omicron variant to be between 32% and 44% ((44)), where asymptomatic rates tend to be higher in younger age groups ((44–46)). Our high asymptomatic rates could be due to reporting errors. In some instances (Supplemental material D) participants reported asymptomatic infection on February 22 and symptomatic infection on February 25, which indicates a possible confusion between asymptomatic and pre-symptomatic infections. Infections asymptomatic at the time of the testing, but with symptoms appearing some days later, should have been reported as symptomatic, but may have been reported as asymptomatic instead, which would lead to an overestimation of the percentage of asymptomatic infections. On the other hand, the survey was conducted two weeks after the RATs were taken, such that participants were given enough time to realize whether symptoms occurred during the infectious period, and correctly report whether infections were symptomatic or not. It could be possible that asymptomatic rates in children and youth are effectively high or that the estimate is unreliable due to low sample size.

The RAT survey results also allowed us to investigate the spatial distribution of COVID-19 cases. We found high heterogeneity in the percentage of positive cases reported across the province, and no relationship between regional proximity and COVID-19 prevalence. Although a positive correlation between COVID-19 prevalence and population density may have been expected ((47,48)), we find that Eastern Health, the RHA with the highest population density, reported the lowest infection prevalence. Due to our small sample size, we could not determine whether the low counts registered for Eastern Health are an artifact of higher reporting rates, and whether using a finer spatial scale or having a larger dataset for certain FSAs could have revealed more insights into the spatial pattern of cases. Previous studies have also found marked heterogeneity in the spatial distribution of COVID-19 cases ((49,50)), where household size, rather than population density, has been recognized to be a better indicator of COVID-19 hotspots ((51,52)).

Given the low participation rate in the survey (3.5%) and small sample sizes, and given that participation in the survey was voluntarily, our results may suffer from sample selection biases, and should be interpreted with caution. It may be that those households with positive tests were more likely to report results, which may have inflated positive case counts in comparison to provincial estimates. Additionally, different social and psychological stresses may have resulted in certain social groups (such as pro or anti-vaccine groups) being more likely to report results than others, leading to additional biases. Finally, sources of bias also occur in the provincial testing system; for example, higher testing rates of vulnerable individuals, hospital admissions and long-term care residents, many of whom are elderly.

## Conclusion

Our analysis of reported data on extensive SARS-CoV-2 testing in NL reveals possible pattern of BA.1/BA.2 prevalence among children and youth, a currently understudied population. We found that in February 2022 only one out of every 4.3 (95% CI, 3.1–5.3) positive households were captured by provincial case count, with asymptomatic infections being 59.8% of the positive cases. Given the low survey participation rate, our results should be interpreted with caution. Nonetheless, our study provides an overview on the epidemiological situation in NL at the time the tests were conducted and discusses the difficulty in obtaining epidemiological data in the context of volatile public health care measures and rampant disease spread.

## Supplemental material

These documents can be accessed on the Supplemental material file.
Supplemental material A: Newfoundland and Labrador provincial case counts
Supplemental material B: Survey
Supplemental material C: Sensitivity and specificity
Supplemental material D: Data handling
Supplemental material E: Estimating underreporting from COVID-19 Immunity Task Force serology data
